# Microstructure, Mechanical Properties, and Corrosion Behavior of Boron Carbide Reinforced Aluminum Alloy (Al-Fe-Si-Zn-Cu) Matrix Composites Produced via Powder Metallurgy Route

**DOI:** 10.3390/ma14154315

**Published:** 2021-08-02

**Authors:** M. Meignanamoorthy, Manickam Ravichandran, Vinayagam Mohanavel, Asif Afzal, T. Sathish, Sagr Alamri, Sher Afghan Khan, C. Ahamed Saleel

**Affiliations:** 1 Department of Mechanical Engineering, K. Ramakrishnan College of Engineering, Trichy 621112, India; mkmmoorthy1990@gmail.com; 2 Centre for Materials Engineering and Regenerative Medicine, Bharath Institute of Higher Education and Research, Chennai 600126, India; mohanavel2k16@gmail.com; 3 P. A. College of Engineering, Visvesvaraya Technological University, Belagavi, Mangaluru 574153, India; 4 Department of Mechanical Engineering, Saveetha School of Engineering, SIMATS, Chennai 602105, India; sathish.sailer@gmail.com; 5 Department of Mechanical Engineering, College of Engineering, King Khalid University, P.O. Box 394, Abha 61421, Saudi Arabia; salamri@kku.edu.sa (S.A.); ahamedsaleel@gmail.com (C.A.S.); 6 Department of Mechanical Engineering, The University of Akron, Akron, OH 44325, USA; 7 Department of Mechanical Engineering, Faculty of Engineering, International Islamic University, Kuala Lumpur 53100, Malaysia; sakhan06@gmail.com

**Keywords:** Al-Fe-Si-Zn-Cu, B_4_C, powder metallurgy, mechanical properties, corrosion

## Abstract

In this paper, Al-Fe-Si-Zn-Cu (AA8079) matrix composites with several weight percentages of B_4_C (0, 5, 10, and 15) were synthesized by powder metallurgy (PM). The essential amount of powders was milled to yield different compositions such as AA8079, AA8079-5 wt.%B_4_C, AA8079-10 wt.%B_4_C, and AA8079-15 wt.%B_4_C. The influence of powder metallurgy parameters on properties’ density, hardness, and compressive strength was examined. The green compacts were produced at three various pressures: 300 MPa, 400 MPa, and 500 MPa. The fabricated green compacts were sintered at 375 °C, 475 °C, and 575 °C for the time period of 1, 2 and 3 h, respectively. Furthermore, the sintered samples were subjected to X-ray diffraction (XRD) analysis, Energy Dispersive Analysis (EDAX), and Scanning Electron Microscope (SEM) examinations. The SEM examination confirmed the uniform dispersal of B_4_C reinforcement with AA8079 matrix. Corrosion behavior of the composites samples was explored. From the studies, it is witnessed that the rise in PM process parameters enhances the density, hardness, compressive strength, and corrosion resistance.

## 1. Introduction

Metal based composites have extensive uses in numerous engineering areas owing to its extreme properties, namely superior precise stiffness, strength/weight ratio, and wear opposition [1]. Traditionally PM has been recognized as suitable method to synthesize metal parts with uniform and fine microstructures. By following this method (PM) various kinds of materials can be easily mixed to attain unique properties [2]. PM is a frequent and fast developing technology, taking up all metallic and alloy materials and a widespread variability of dimensions [3]. Compared to other conventional fabrication methods, the PM route is recognized to be capable in the fabrication of aluminum alloy based MMC [4]. Due to the collective effect of metallic and ceramic materials, aluminum metal matrix composites have tremendous uses such as automotive and aircraft, owing to its low density and specific strength [5]. Aluminum alloy has numerous merits compared to Fe alloys, namely lower density, higher conductivity, etc. [6]. Among various reinforcements, boron carbide (B_4_C) has been accepted one of the hardest. B_4_C possesses better wear and impact opposition, a higher maximum melting point, and better resistance to chemical agents [7,8]. B_4_C has been extensively utilized as cements and armor plates, despite its less specific gravity, better hardness value, and maximum elastic modulus value [9]. Hamid Alihosseini et al. inspected the behavior and microstructure analysis of B_4_C/Al nanocomposites produced through the PM method, and stated that maximum hardness and compression strength was achieved for Al-5% B_4_C composites [10]. Jeyasimman et al. [11] examined the microstructure and mechanical behavior of AA6061-γ-Al_2_O_3_ nanocomposites produced through mechanical alloying and PM route and described the mechanical properties. Anil Kumar Bodukuri et al. synthesized B_4_C/SiC/Al powder metallurgic composites and studied the mechanical behavior [12]. Sivasankaran et al. produced and investigated the x-ray diffraction of Al_2_O_3_ reinforced nanocomposite manufactured via mechanical alloying [13]. Ravichandran et al. explored the microstructure and EDAX analysis of Al–TiO_2_–Gr composites, and observed the presence and dispersal of reinforcement particles with matrix [14]. Mohammed Ali Almomani et al. studied the corrosion properties of Cu-30Zn Brass with and without SiC reinforcement, fabricated through powder metallurgy, and stated that corrosion opposition of the composites enhanced the raise of percentage as an effect of weedy micro galvanic combination amid reinforcement particles and alloy [15]. H.M. Zakaria explored the microstructure and conduct of SiC strengthened Al composites manufactured through PM method and observed that addition of reinforcement leads to reduction in corrosion rate [16].

Norul AmierahBinti Nor Zamani et al. [17] studied the mechanical characterization of Al+Gr+Al_2_O_3_ hybrid composites and stated that inclusions of Al_2_O_3_ and Gr particles improve the mechanical behavior of the AMCs considerably. Nazli Akcamli [18] developed Al-8.5 wt% Si-3.5 wt% Cu matrix composites and reported that inclusion of B_4_C particles results in significant enhancement in mechanical behavior of the produced composites. Vipin Kumar Sharma [19] synthesized Al6061-Al_2_O_3_-SiC-CeO_2_ composites by PM route and concluded that a rise in reinforcement wt.% enhances the composites mechanical behavior drastically. Mohd Bilal Naim Shaikh [20] explored the mechanical behavior of Al-SiC-RHA composites produced via the PM method and observed that inclusions of reinforcement particles result in superior improvement in mechanical properties of the composites. Meysam Toozandehjani et al. [21] produced Al-CNT-Al_2_O_3_ nanocomposites through the PM process and observed that superior hardness and strength properties were attained at integration of 10 wt.% Al_2_O_3_. Erdemir et al. [22] produced Al2024/SiC composites via the PM process and indicated that inclusions of SiC reinforcement enhances the mechanical properties of the composites expressively. Halil Karakoc et al. [23] explored the mechanical properties of Al6061/SiC/B_4_C hybrid composites prepared by the PM route, and concluded that increase in reinforcement particles results in enhancement in mechanical properties. Fathy et al. [24] explored the mechanical properties of Al-Fe composites and stated that the addition of Fe reinforcement particles increases the hardness and compressive strength of the composites Gheorghe Iacob et al. [25] investigated the micro hardness behavior of Al/Al_2_O_3_/Gr hybrid composites produced via the PM process and witnessed that a rise in reinforcement weight percentage increases the micro hardness gradually. Stalin et al. [26] studied the corrosion behavior of Al-MoO_3_ composites synthesized by the PM route; from the experimentation it has been concluded that corrosion opposition of the composites enhanced with the addition of a MoO_3_ particle.

From the detailed literature review, it could be understood that very little research work has been completed in the development of aluminum alloys of 8xxx series using PM technique. Additionally, present work aims to develop composite with the AA8079 and B_4_C particle. From the literature review it has been found that the development of aluminum alloy is a challenging task. Hence, ball milling was used to develop aluminum alloy in the present work. However, the mixing of alloying element and reinforcement particle with the major constituent could be possible by selecting suitable ball milling and powder metallurgy process parameters. Furthermore, this work has made an effort to synthesize AA8079-B_4_C composites at different powder metallurgy process parameters to analyze the microstructure, mechanical, and corrosion behavior. The effect of parameters on the hardness, density, CS, corrosion properties, and microstructure have been analyzed in detail.

## 2. Experimental Details

**Figure 1:**
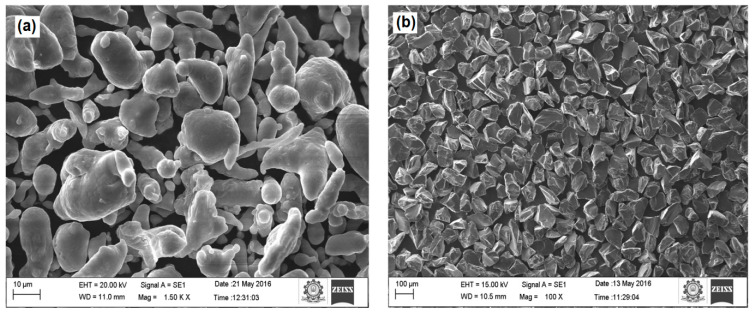
SEM images of the as-received (**a**) Al and (**b**) B_4_C.

AA8079 was manufactured via mixing the 99.5% elemental powders aluminum (100 μm), copper (10 μm), iron (10 μm), silicon (8 μm), and zinc (10 μm). Boron carbide of size 10 μm was utilized as reinforcement. The aluminum and B_4_C was purchased from kemphasol, Mumbai, India. The other powders, such as copper, iron, silicon, and zinc were purchased from Lobachemi, Mumbai, India. SEM image of the as procured Al and B_4_C powders are displayed in Figure 1a,b.

**Figure 2:**
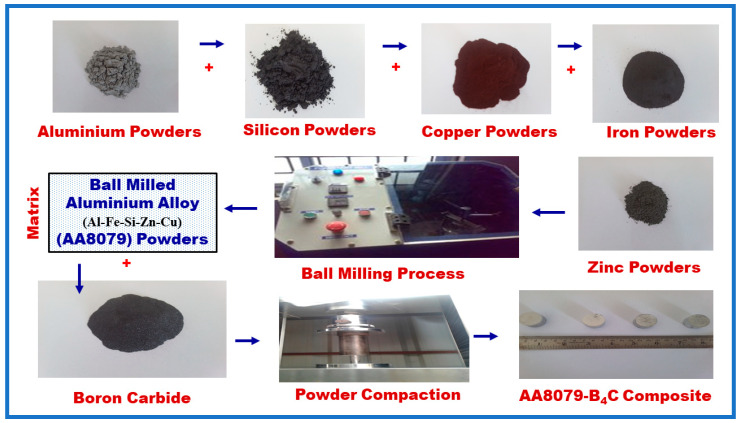
Synthesis of AA8079 and composite samples using ball milling and compaction method.

The composite powders were synthesized using high energy ball mill for 10 h (VBCRC Planetary ball mill). The drum speed was 100 rpm. A steel ball with 10 mm diameter was used. The ball to powder ratio was 5:1. To avoid the temperature rising, a cooling process was carried out every 10 min as per [27]. The green compacts were made into billets of dimensions 24 mm diameter and 12 mm height using a computer servo-controlled ball screw driven UTM (Model: M Series). To avoid the friction between the punch and die, zinc stearate was used as lubricant. Figure 2 shows the details of powders and ball milling setup.

**Figure 3:**
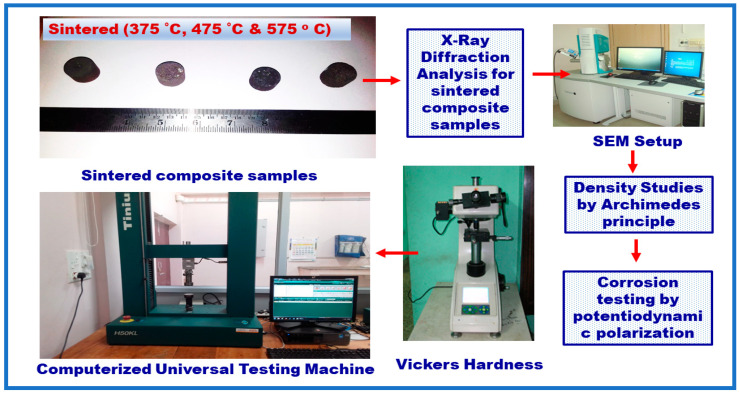
Characterization and mechanical properties studies for the sintered composite samples.

Then, the green compacts were sintered at various temperatures of 375 °C, 475 °C, and 575 °C for a period of 1, 2, and 3 h, respectively, using electric muffle furnace (HITECH India) below controlled atmosphere to evade oxidation; the sintered samples were retained in the furnace until it reaches the room temperature [28]. By following the rule of mixture, the sintered density was measured for all the samples by Archimedes principle. Three readings were measured, and their average value was taken [29]. XRD analysis was accompanied on the Al and B_4_C sintered preforms to study the phase identification using X-ray diffractometer (Broker Eco D 8). The chemical compositional examination was examined via energy dispersive analysis (via EDAX-AMETEK-TSL). Figure 3 shows the details of testing conducted for the composite samples.

The microstructure analysis of sintered composite samples was performed by SEM (ZESIS model). The micro Vickers hardness test was carried out using micro Vickers hardness tester (Model: MV-1 PC), test was carried out at a load of 0.3 kg and a stay time of 10 s, as per ASTM standard E384-08. The compressive test was carried using computer controlled universal testing machine (Tinius Olsen) having a capacity of 50 kN in accordance with ASTM standard E9-89a. Electrochemical measurement was achieved by utilizing Versa STAT MC. Later, for polarization examinations, electrodes were utilized for the electrochemical impedance spectroscopy (EIS) examination deprived of any surface treatment. The AA8079/B_4_C composite samples with 1.0 cm^2^ surface area are wide-open to corrosion medium of 3.5% NaCl solutions. The potentio-dynamic current–potential curves were obtained by polarizing the specimen from −0.1 V to +0.1 V on open circuit potential at a scan rate of 0.05 mV/s. EIS measurements was conducted utilizing a small amplitude AC signal of 10 Mv over a frequency of 100 kHz–0.01 Hz [30,31]. The microstructures of samples after compression test were examined utilizing the SEM.

## 3. Results and Discussions

This section explains the microstructure and characterization studies of sintered composite preforms, and the effect of PM parameters on the density, hardness, CS, corrosion behavior, and the microstructure of the composites after compression test.

### 3.1. Characterization Studies on Sintered Preforms

**Figure 4:**
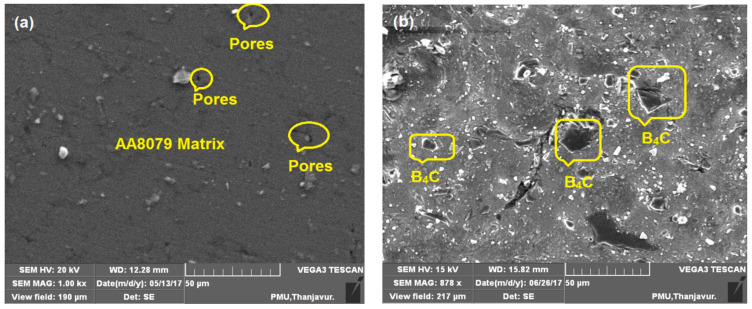

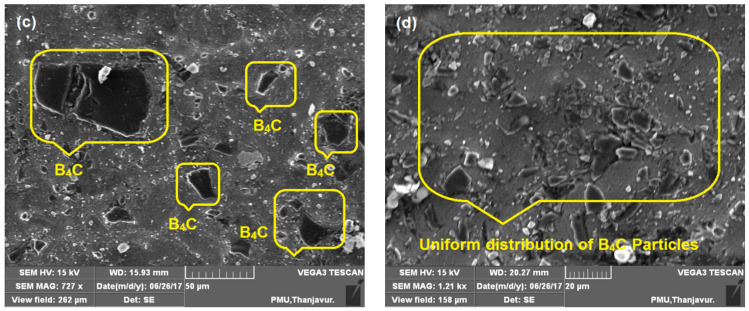
SEM images of sintered (**a**) AA8079, (**b**) AA8079-5 wt.%B_4_C, (**c**) AA8079-10 wt.%B_4_C, and (**d**) AA8079-15 wt.%B_4_C composite preforms.

The microstructure analysis of the as-sintered AA8079, AA8079-5 wt.%B_4_C, AA8079-10 wt.%B_4_C, and AA8079-15 wt.%B_4_C composites preforms has been studied using SEM. Figure 4a displays the microstructure of AA8079; it ensures the absence of B_4_C content, and it can be seen that pores are witnessed for unreinforced AA8079. Furthermore, no pore has been found in the composite preforms, due to occupation of B_4_C particle in the matrix. Figure 4b displays the SEM micrograph of composite contains 5 wt.% of B_4_C and the presence of B_4_C particles are evident.

From Figure 4c, the uniform distribution of B_4_C particle can be seen, and no pores or crack has been found. Enhanced interfacial connection was attained amid the matrix and B_4_C particles. Figure 4d displays the SEM image of composite containing 15 wt.% of B_4_C, and from the image no agglomeration of particles was observed.

Some researchers reported agglomeration issues for the inclusion of 10 wt.% of B_4_C in Al matrix. However, in this work, we overcome that problem by selecting the suitable ball milling parameters [32,33,34]. Due to higher pressure amid the compaction, a dense microstructure was acquired which was supportive in material strength enhancement, with fine distribution of reinforcement with matrix. Particles were combined and filled closely with matrix which enhanced the mechanical properties. The separation of B_4_C with matrix is also of note. The SEM revealed the occurrence of the distributed phase, which is the B_4_C is dispersed evenly in the matrix.

**Figure 5:**
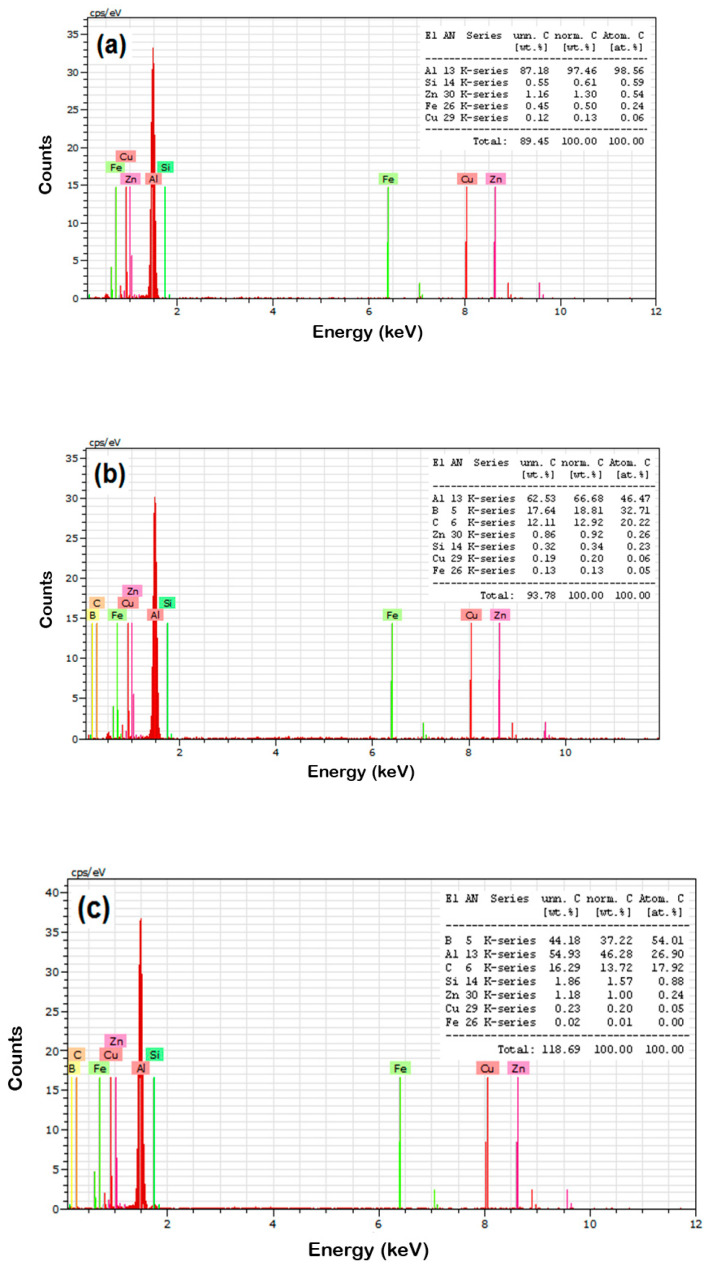

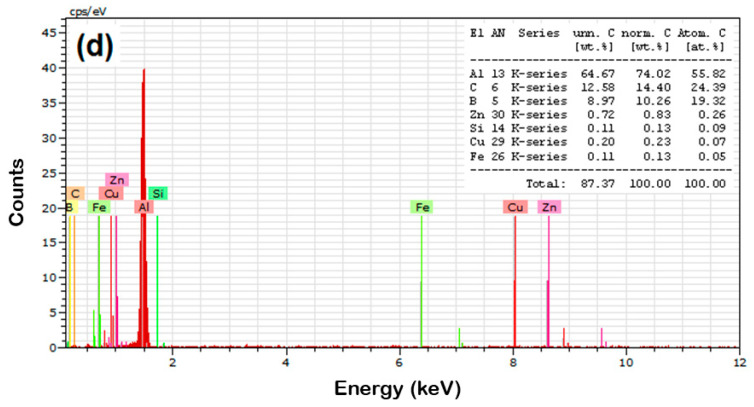
EDAX of sintered (**a**) AA8079, (**b**) AA8079-5% B_4_C, (**c**) AA8079-10% B_4_C, and (**d**) AA8079-15% B_4_C composite preforms.

The EDAX analysis of the sintered composites preforms are displayed in Figure 5. Figure 5a displays the occurrence of Al peaks with high intensity, and Cu, Fe, Si, and Zn peaks with very low intensities. Figure 5b–d displays the existence of Al peaks with great intensity, and B, C, Cu, Fe, Si, and Zn peaks were also observed for composite samples. The results show that elemental and reinforcement particles were homogenously dispersed with the aluminum matrix due to the proper milling, compaction, and sintering process. From this analysis it is obvious that the occurrence of respective elements of alloy and composite samples is evident. It is clear that 5 to 15 wt.% of B_4_C was predicted with fine dispersal with matrix.

**Figure 6:**
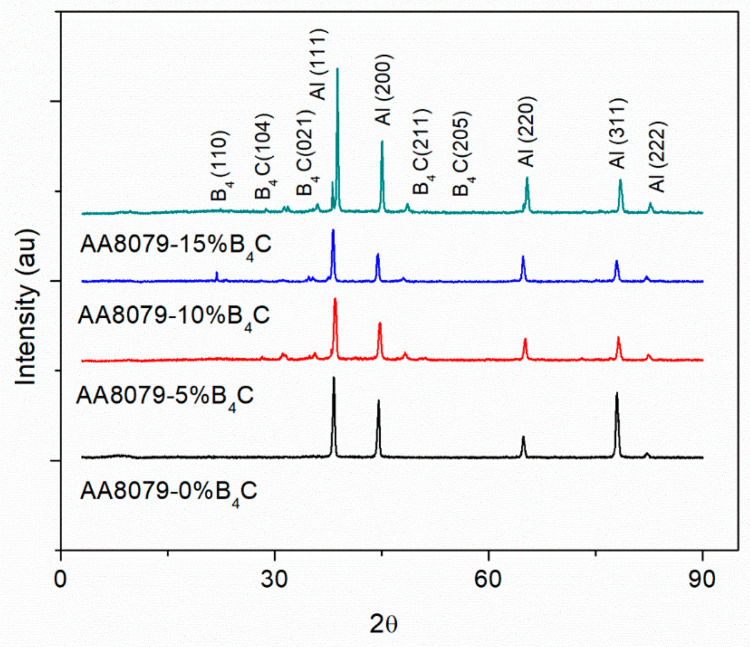
XRD patterns of AA8079-B_4_C composites.

Figure 6 noticeably shows the XRD patterns of preforms sintered at 575 °C. Amid the various compounds identified, aluminum influenced the strongest peak, and it ensures the Al is the major content in this material. The occurrence of B_4_C peaks reveals the occurrence of B_4_C in (110), (104), (021), (211), and (205) planes. The intensity of B_4_C peaks enhances with the raise in weight percentage of B_4_C in the composites. XRD results confirm the occurrence of aluminum in the major peak, and the occurrence of B_4_C, exposed by small peak, and it ensures the respective weight percentage of the composites. Furthermore, it has been confirmed that no intermetallic compounds were formed during the sintering process as reported by the previous researchers [35,36]. Peaks for Fe, Si, Zn, and Cu interrelated to the AA8079 were not witnessed due to the development of a solid solution.

### 3.2. Effect of PM Parameters on Density

**Figure 7:**
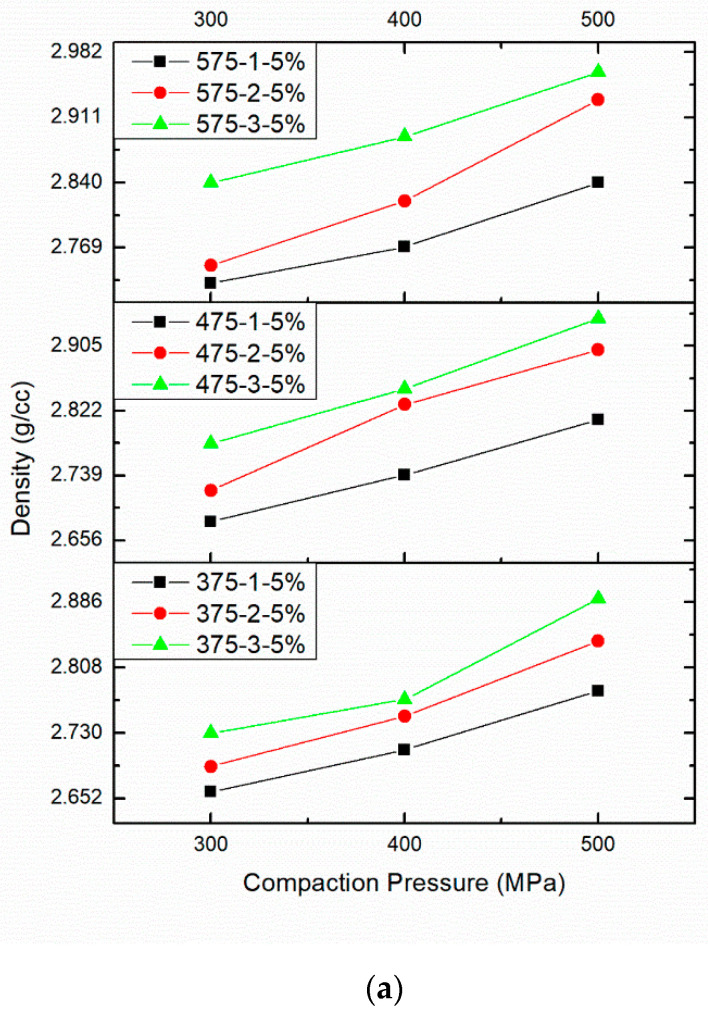

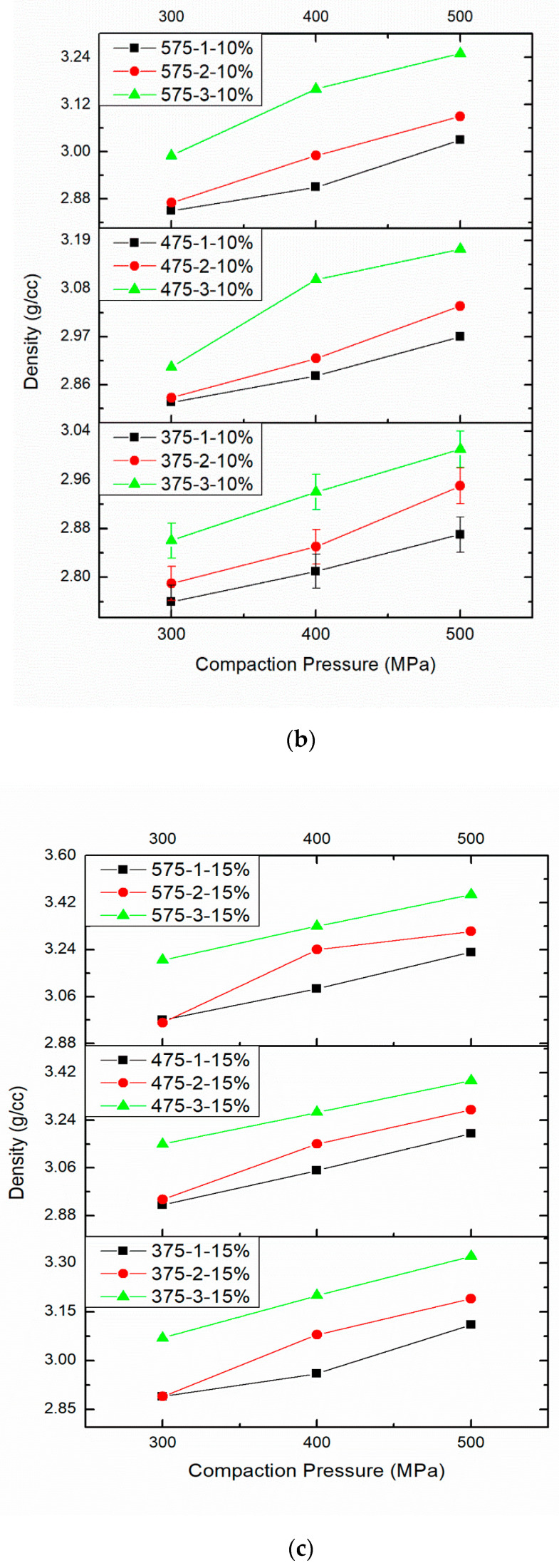
(**a**). Effect of different compaction pressure (300, 400, and 500 MPa), different sintering time (1, 2, and 3 h), sintering temperature (375 °C, 475 °C, and 575 °C), and 5 wt%.B_4_C on density. (**b**) Effect of different compaction pressure (300, 400, and 500 MPa), different sintering time (1, 2, and 3 h), sintering temperature (375 °C, 475 °C, and 575 °C), and 10wt%.B_4_C on density. (**c**) Effect of different compaction pressure (300, 400, and 500 MPa), different sintering time (1, 2, and 3 h), sintering temperature (375 °C, 475 °C, and 575 °C) and 15 wt%.B_4_C on density.

The influence of compaction pressure (CP), sintering temperature (S.Temp) and sintering time (ST) on the density are provided in Figure 7a–c. Table 1 provides the effect of PM parameters on density. Figure 7a shows the density of AA8079-5 wt.%B_4_C composites with respect to CP, S.Temp, and ST. The increase in CP, S.Temp, and ST increases the density of the AA8079-5 wt.%B_4_C composites. For AA8079-5 wt.%B_4_C composites, maximum density of 2.96 g/cm^3^ was attained at CP of 500 MPa, S.Temp of 575 °C, and ST of 3 h. Densification is proportional to CP, S.Temp, and ST. The rate of dispersal enhances, while increase in S.Temp offers good densification at high temperature.

Figure 7b shows the density for the AA8079-10 wt.%B_4_C composites. The increase in CP, S.Temp, and ST increases the density of the AA8079-10 wt.%B_4_C composites. For AA8079-10 wt.%B_4_C composites, maximum density of 3.25 g/cm^3^ was obtained at CP of 500 MPa, S.Temp of 575 °C, ST of (1, 2, and 3 h). Enhancement in ST offers much time for pore closure in the matrix; henceforth, densification is perceived to rise with the rise in ST. At 575 °C, the density enhances due to a decrease in pores. Amid the sintering process, reduction in the samples occurs, despite the volume of diffusion of atoms from the grain boundary sources to the voids, which results in density enhancement.

Figure 7c shows the density for the AA8079-15 wt.%B_4_C composites. The increase in CP, S.Temp, and ST increases the density of the AA8079-15 wt.%B_4_C composites. For AA8079-15 wt.%B_4_C composites, maximum density of 3.45 g/cm^3^ was obtained at CP of 500 MPa, S.Temp of 575 °C, and ST of 3 h. It could be understood that an increase in CP, S.Temp, and ST enhances the density of the AA8079-B_4_C composites. This is due to the fact that diffusion of particles and decrease in porosity occurred; it results in improved density as reported by Patel et al. [27]. Generally, the current investigation stated that to fabricate AA8079-B_4_C composites at a maximum density, the specimen would be compacted to 500 MPa and sintered at 575 °C temperature for 3 h.

### 3.3. Effect of PM Parameters on Micro Vickers Hardness

**Figure 8:**
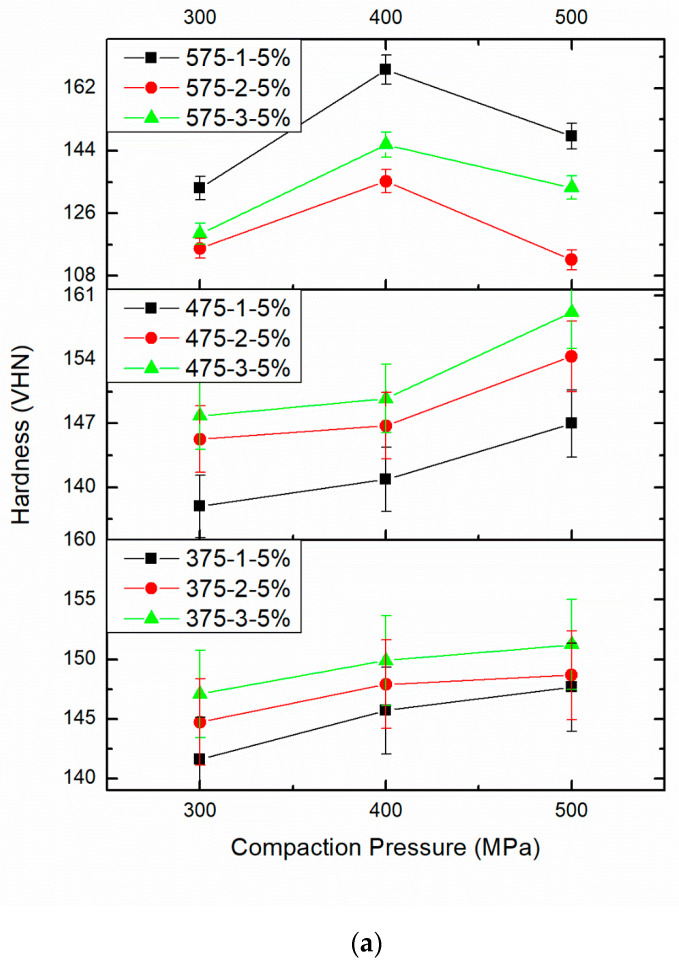

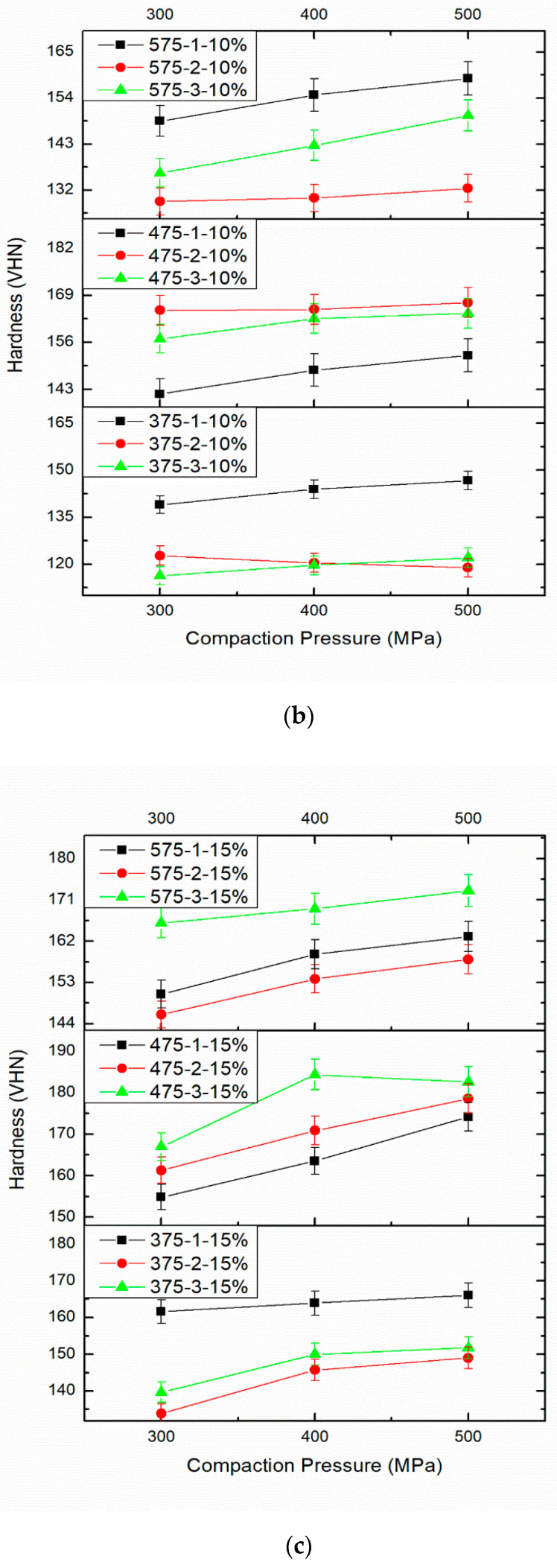
(**a**). Effect of different compaction pressure (300, 400, and 500 MPa), different sintering time (1, 2, and 3 h), sintering temperature (375 °C, 475 °C, and 575 °C), and 5 wt%.B_4_C on micro hardness. (**b**). Effect of different compaction pressure (300, 400, and 500 MPa), different sintering time (1, 2, and 3 h), sintering temperature (375 °C, 475 °C, and 575 °C), and 10wt%.B_4_C on micro hardness. (**c**). Effect of different compaction pressure (300, 400, and 500 MPa), different sintering time (1, 2, and 3 h), sintering temperature (375 °C, 475 °C, and 575 °C), and 15 wt%.B_4_C on micro hardness.

Figure 8a–c displays the influence of PM parameters on the hardness of AA8079-B_4_C composites with respect to CP, S.Temp, and ST. Table 2 provides the effect of PM parameters on micro hardness. The maximum hardness is witnessed for the specimens compacted at 500 MPa, and sintered at 575 °C for 3 h. From Figure 8a–c, the hardness of specimens improves whereas enhancing the CP from 300 MPa to 500 MPa, S.Temp from 375 °C to 575 °C, and ST from 1 h to 3 h. At CP greater than 500 MPa, the applied loads force the particles to transfer, blending with one another and blocking the voids, henceforth attaining maximum hardness for AA8079-B_4_C composites. Increasing the CP, S.Temp, and ST results in hardness enhancement due to maximum densification. When the PM process parameters increased, pores and voids present in the samples were completely occupied by the B_4_C particles. This could be one of the reasons for hardness increment.

The applied load results in particle deformation; however, alterations in particle size and shape improve the hardness. The hardness upsurges while enhancing the CP, S.Temp, and ST. It is obvious that when the CP, S.Temp, and ST increase, the hardness of the composites enhances due to particle-to-particle appropriate bonding. Furthermore, B_4_C is the third hardest material, due to the fact that the hardness of the composites increased gradually. These outcomes are all around concurred with the earlier findings of different researchers [7,8,37,38]. When the samples prepared at maximum PM process parameters at that time grain refinement and proper dispersal of B_4_C with AA8079 occurred, it resulted in maximum hardness enhancement. At maximum sintering temperature, particle-to-particle binding takes place, forming a better bond by the diffusion of atoms in a solid-state bonding method. Improved ductility, dispersion strengthening mechanism, and refinement of grain size result in enhanced hardness [39,40,41].

### 3.4. Effect of PM Parameters on Compressive Strength

**Figure 9:**
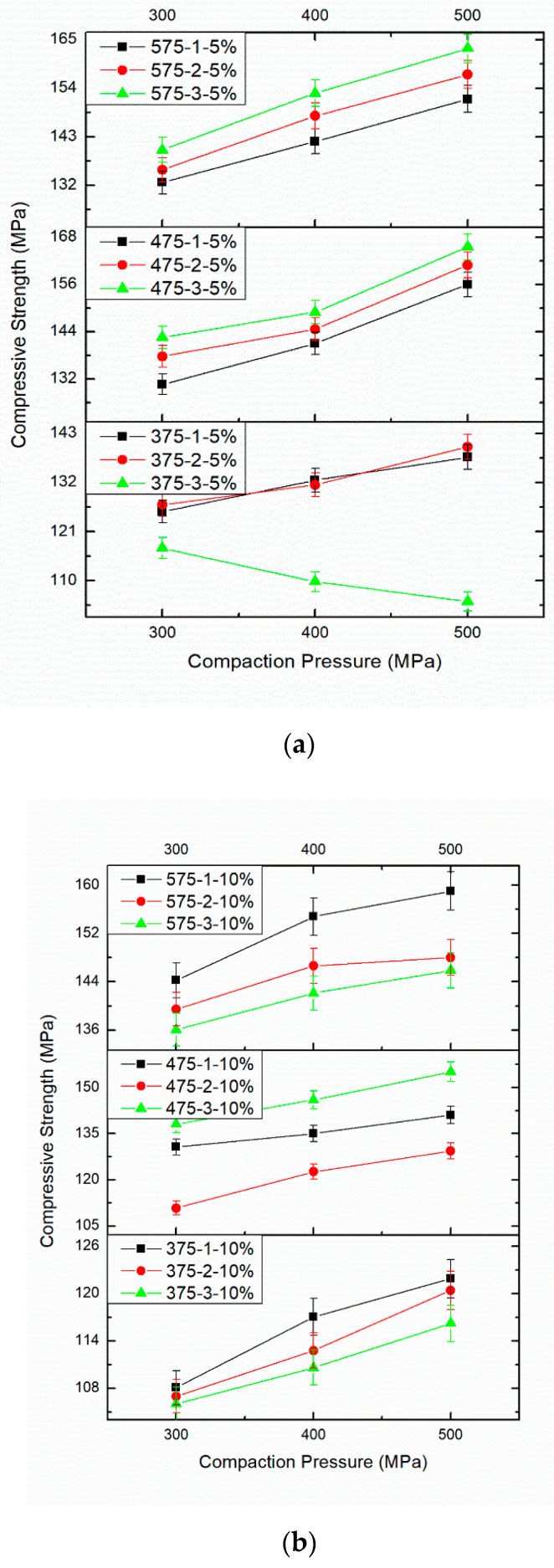

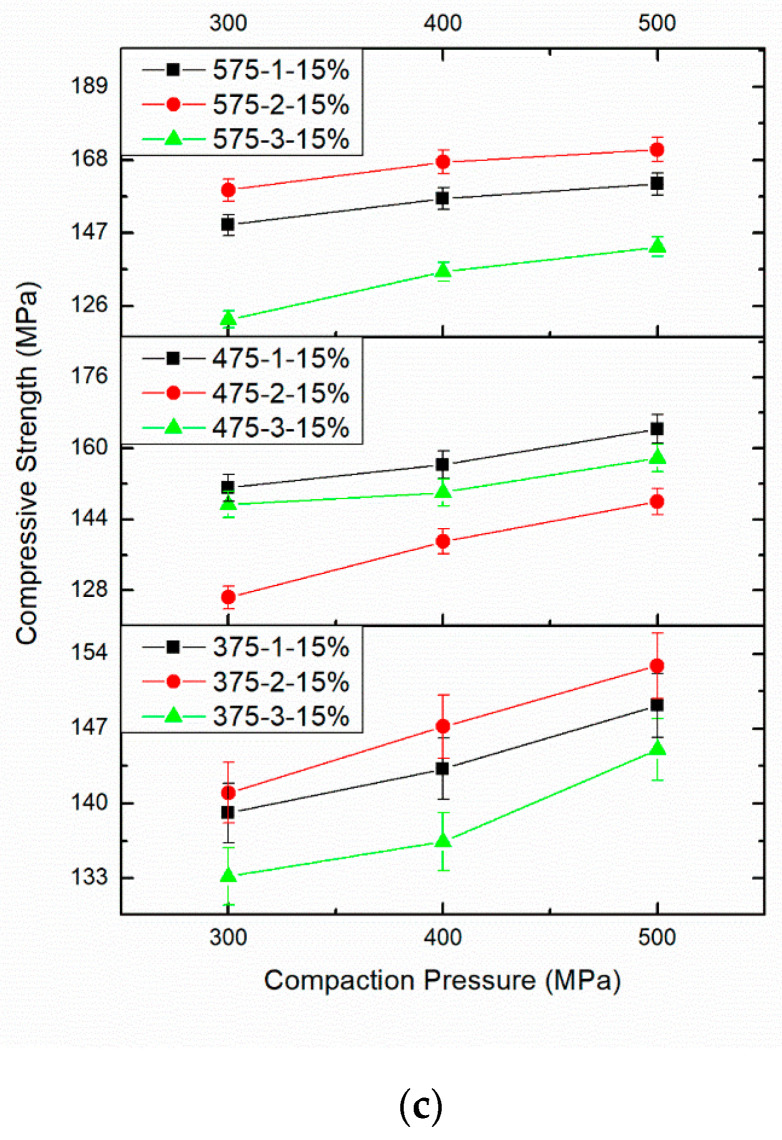
(**a**). Effect of different compaction pressure (300, 400, and 500 MPa), different sintering time (1, 2, and 3 h), sintering temperature (375 °C, 475 °C, and 575 °C), and 5 wt%.B_4_C on compressive strength. (**b**). Effect of different compaction pressure (300, 400, and 500 MPa), different sintering time (1, 2, and 3 h), sintering temperature (375 °C, 475 °C, and 575 °C), and 10wt%.B_4_C on compressive strength. (**c**)**.** Effect of different compaction pressure (300, 400, and 500 MPa), different sintering time (1, 2, and 3 h), sintering temperature (375 °C, 475 °C, and 575 °C), and 15 wt%.B_4_C on compressive strength.

Figure 9a–c displays the influence of PM parameters on the compressive strength of AA8079-B_4_C composites with respect to CP, S.Temp, and ST. Table 3 provides the effect of PM parameters on compressive strength. The maximum compressive strength is perceived for the specimens compacted at 500 MPa, sintered at 575 °C for 3 h. From Figure 9a–c, the compressive strength of specimens improves, enhancing the CP from 300 MPa to 500 MPa, the S.Temp from 375 °C to 575 °C, and the ST from 1 h to 3 h. From this, it is observed that the rise in S.Temp and ST increases the compressive strength. It is understood that the upsurge in CP, S.Temp, and ST enhances the compressive strength. This could be elucidated through the way that a rise in CP, S.Temp, and ST improves the heat treatment method by which appropriate holding and dissemination of particles is accomplished [42,43,44,45,46].

Additionally, the compressive strength of the composites totally relies upon the PM parameters, which create the enhancement in properties conceivable. As indicated by this examination, the most elevated compressive strength was noticed for the specimen compacted at 500 MPa, and sintered at 575 °C for 3 h. The enhancement in the compressive strength may be accredited to the shifting of load from matrix to the hard reinforcement [47,48]. The increasing strength of these composites as the B_4_C wt.% rises could be ascribed to the dispersal strengthening effect [49]. The maximum plastic deformation and strain hardening acquaint with powder amid compaction at maximum pressure to produce good results, leading to maximum compressive strength. The enhancement in loading resistance enhances the compressive strength [50]. Higher plastic deformation and strain hardening introduced in the powder during compaction at higher pressures yield better results and contributed to higher compression strength [51].

### 3.5. Microstructure Analysis of Specimens after Compression Test

**Figure 10:**
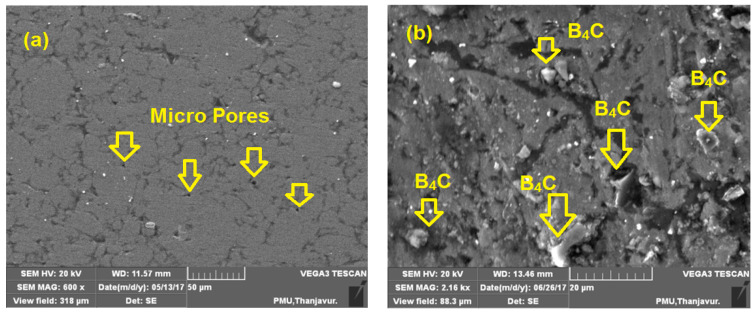

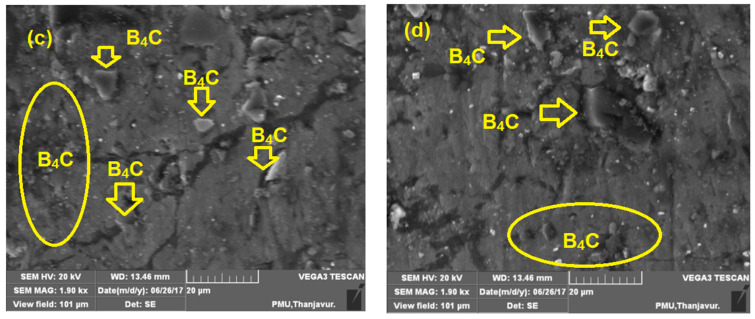
SEM images after compression test (**a**) 0%, (**b**) 5% B_4_C, (**c**) 10% B_4_C, and (**d**) 15% B_4_C samples.

The microstructure of the preforms after the compression test are shown in Figure 10a–d. The observable large pore sizes in sintered samples are reduced in the AA8079 matrix after compression testing. During the compression test the compressive load improved the microstructure of the produced powder metallurgy materials. Furthermore, no pores were found in the composite samples. After the compression test, grain boundaries are elongated due to deformation of the samples, and the hard ceramic particles are finely covered by the matrix materials due to deformation. Figure 10b,c displays the homogenous distribution of B_4_C particle into the matrix alloy. It is clear from the SEM images in Figure 10b–d that virtuous interfacial bond occurs amid the AA8079 and boron carbide. Due to the appropriate compressive force applied over the samples, the particles are distributed evenly within the matrix. Furthermore, it is witnessed that B_4_C particles are reoriented in the way of metal flow during compression process.

### 3.6. Corrosion Behavior

**Figure 11:**
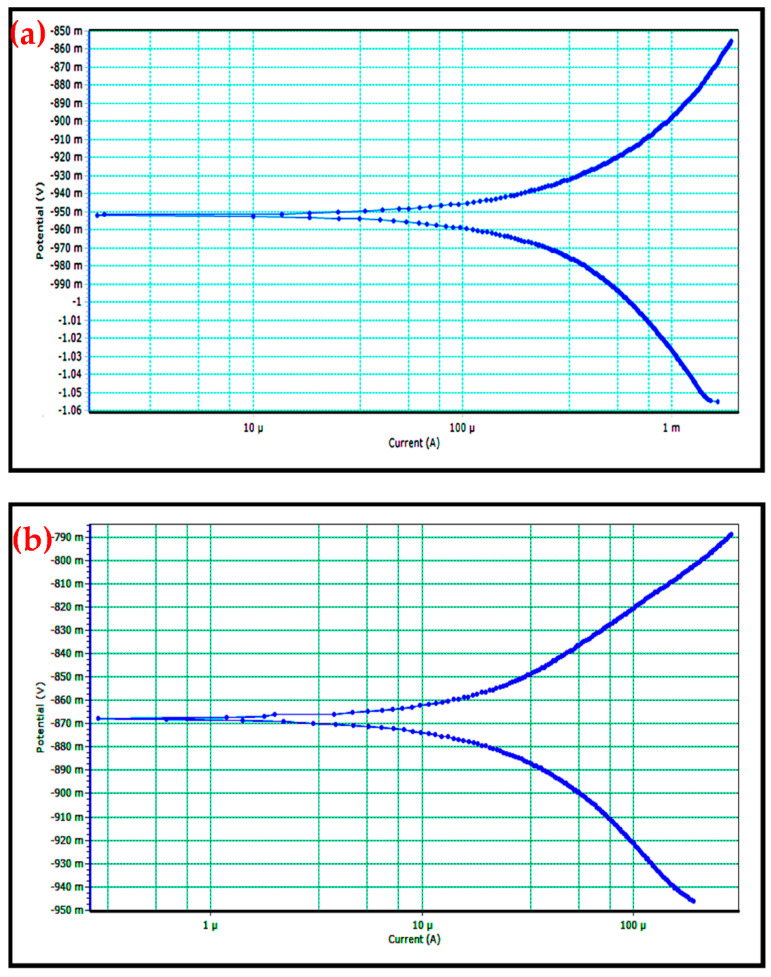

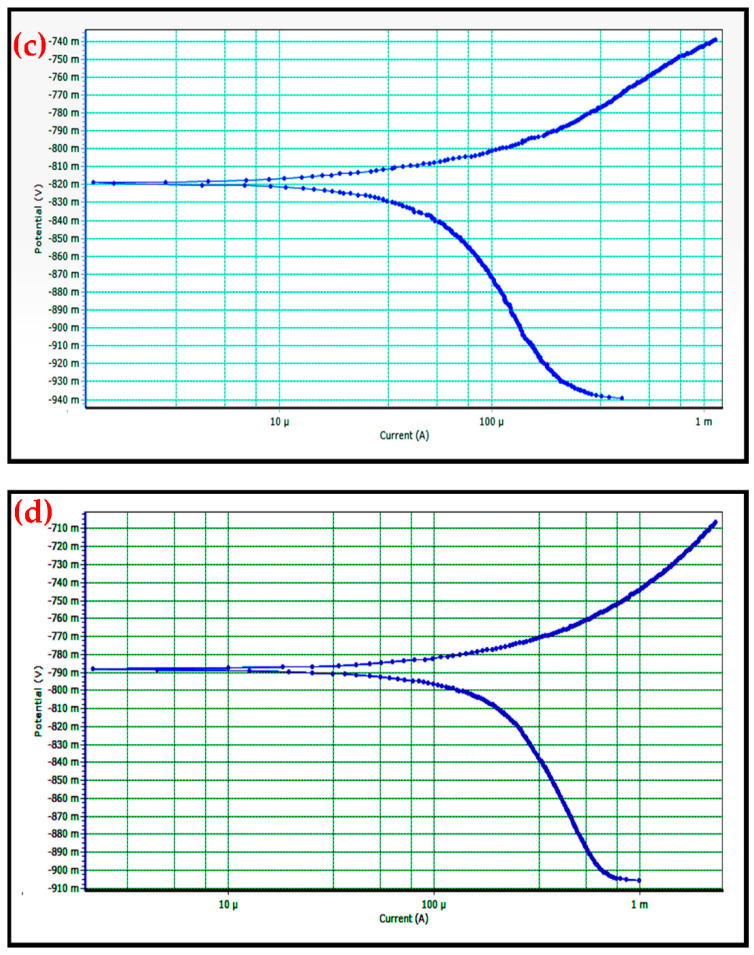
(**a**–**d**) Polarization curves for (**a**) AA8079, (**b**) AA8079-5 wt.%B_4_C, (**c**) AA8079-10 wt.%B_4_C, and (**d**) AA8079-15 wt.%B_4_C composites in 3.5% NaCl solution.

The corrosion behavior of composite samples has been studied by using electroanalytical techniques such as polarization and impedance measurements (EIS). The polarization curve of samples are displayed in Figure 11a–d. Tafel plots indicate that the corrosion rate of the composites reduced with raising the B_4_C weight percentage. Hence, galvanic influence amid them is detached. The witnessed increase in corrosion resistance for composites is dispensed to probable electrochemical decoupling between B_4_C particles and AA8079 matrix [15]. Soorya Prakash et al. reported that corrosion resistance rises considerably with a rise in hard particulate reinforcement such as B_4_C [52]. In inorganic acid forms, corrosion rate enhances as polarization curves are moved to a higher current density area associated to neutral chloride forms. B_4_C particles perform as physical protectors to stop the actuation and rate of development for pitting corrosion. The anodic polarization curves for AA8079 and AA8079-B_4_C display the endurance in corrosion current density, representing the exposure of pitting corrosion. The B_4_C particles which are utilized as reinforcing elements impede the creation of oxide layer and thus reduce the composites corrosion rate expressively.

**Figure 12:**
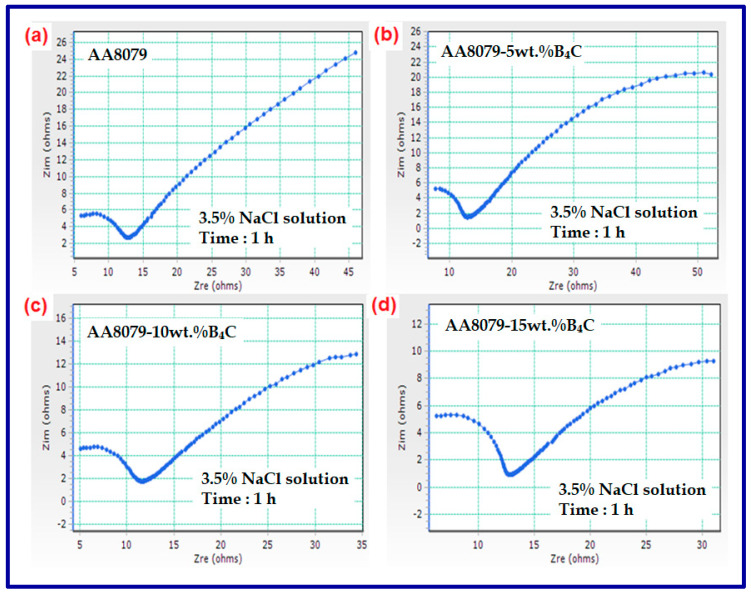
(**a**–**d**) Nyquist plots for (**a**) AA8079, (**b**) AA8079-5 wt.%B_4_C, (**c**) AA8079-10 wt.%B_4_C, and (**d**) AA8079-15 wt.%B_4_C composites in 3.5%NaCl solutions.

The Nyquist plots observed in 3.5% Nacl solution for the AA8079, AA8079-5 wt.%B_4_C, AA8079-10 wt.%B_4_C, and AA8079-15 wt.%B_4_C composites are shown in Figure 12a–d. EIS for all the samples were detected after OCP recorded for 1 h. The occurrence of a defensive oxide film on the layers of composites is despite the attribution of a high frequency capacitive loop. The preforms are occupied through the oxide film capacitance; furthermore, the capacitance arcs diameters increased with an increase in the B_4_C particle; perhaps the opposition of the surface oxide film on the samples upsurges with the enhancement in B_4_C reinforcement. The uneven semicircle displays a non-ideal electrochemical performance on the electrode surface, which is despite the frequency distribution, roughness of the metal surface, and inhomogeneity. The Nyquist plot displays capacitive loop which is linked to the behavior of double layer capacitance, along with the charge transfer process amid electrolyte and metal surface. The diameter of the semicircle decreases with an increase in acid concentration, indicating an increase in the corrosion rate. A rise in the diameters of the Nyquist plots indicates the improved protective nature of the inhibitor against damage of material in the corrosive solution [53]. It could be understood that corrosion resistance increases when increasing the B_4_C weight percentage.

## 4. Conclusions

AA8079 matrix composites containing different weight percentage of B_4_C as reinforcements were successfully fabricated at different PM process parameters, and the subsequent conclusions were obtained:

1. From the SEM examination, fine dispersal and occurrence of B_4_C particles with the AA8079 matrix has been observed;
2. XRD analysis shows the presence of B_4_C particles with minor peaks;
3. The EDAX analysis of the sintered samples witnessed the existence of B_4_C particles with AA8079 matrix and the respective elemental powders of the AA8079 matrix;
4. The density, hardness, and compressive strength of the composite was increased while increasing the reinforcement weight percentage from 5 to 15 wt.% with respect to an increase in PM process parameters, compaction pressure, sintering temperature, and time;
5. The SEM micrographs, after compression testing, exposed the homogenous dispersal of B_4_C reinforcement with AA8079 matrix without pores and grain boundaries;
6. The AA8079-B_4_C composites corrosion resistance rose with a rise in weight percentage of B_4_C reinforcement with AA8079 matrix.

## Figures and Tables

**Table 1:** Effect of PM parameters on density at different compaction pressure (300, 400, and 500 MPa), different sintering time (1, 2, and 3 h), different sintering temperature (375, 475, and 575 °C), and different reinforcement weight percentages (5 wt%., 10 wt%., and 15 wt%.B_4_C).

**1**	**Compaction Pressure** **300 MPa**	**Compaction Pressure** **400 MPa**	**Compaction Pressure** **500 MPa**
	**Sintering Time 1 h**	**Sintering Time 2 h**	**Sintering Time** **3 h**
Sintering Temperature 375 °C and 5 wt%.B_4_C	2.66	2.69	2.73
Sintering Temperature 375 °C and 5 wt%.B_4_C	2.71	2.75	2.77
Sintering Temperature 375 °C and 5 wt%.B_4_C	2.78	2.84	2.89
**2**	**Compaction Pressure** **300 MPa**	**Compaction Pressure** **400 MPa**	**Compaction Pressure** **500 MPa**
Sintering Temperature 475 °C and 5 wt%.B_4_C	2.68	2.72	2.78
Sintering Temperature 475 °C and 5 wt%.B_4_C	2.74	2.83	2.85
Sintering Temperature 475 °C and 5 wt%.B_4_C	2.81	2.90	2.94
**3**	**Compaction Pressure** **300 MPa**	**Compaction Pressure** **400 MPa**	**Compaction Pressure** **500 MPa**
Sintering Temperature 575 °C and 5 wt%.B_4_C	2.73	2.75	2.84
Sintering Temperature 575 °C and 5 wt%.B_4_C	2.77	2.82	2.89
Sintering Temperature 575 °C and 5 wt%.B_4_C	2.84	2.93	2.96
**4**	**Compaction Pressure** **300 MPa**	**Compaction Pressure** **400 MPa**	**Compaction Pressure** **500 MPa**
Sintering Temperature 375 °C and 10 wt%.B_4_C	2.76	2.79	2.86
Sintering Temperature 375 °C and 10 wt%.B_4_C	2.81	2.85	2.94
Sintering Temperature 375 °C and 10 wt%.B_4_C	2.87	2.95	3.01
**5**	**Compaction Pressure** **300 MPa**	**Compaction Pressure** **400 MPa**	**Compaction Pressure** **500 MPa**
Sintering Temperature 475 °C and 10 wt%.B_4_C	2.82	2.83	2.90
Sintering Temperature 475 °C and 10 wt%.B_4_C	2.88	2.92	3.10
Sintering Temperature 475 °C and 10 wt%.B_4_C	2.97	3.04	3.17
**6**	**Compaction Pressure** **300 MPa**	**Compaction Pressure** **400 MPa**	**Compaction Pressure** **500 MPa**
Sintering Temperature 575 °C and 10 wt%.B_4_C	2.85	2.87	2.99
Sintering Temperature 575 °C and 10 wt%.B_4_C	2.91	2.99	3.16
Sintering Temperature 575 °C and 10 wt%.B_4_C	3.03	3.09	3.25
**7**	**Compaction Pressure** **300 MPa**	**Compaction Pressure** **400 MPa**	**Compaction Pressure** **500 MPa**
Sintering Temperature 375 °C and 15 wt%.B_4_C	2.89	2.89	3.07
Sintering Temperature 375 °C and 15 wt%.B_4_C	2.96	3.08	3.20
Sintering Temperature 375 °C and 15 wt%.B_4_C	3.11	3.19	3.32
**8**	**Compaction Pressure** **300 MPa**	**Compaction Pressure** **400 MPa**	**Compaction Pressure** **500 MPa**
Sintering Temperature 475 °C and 15 wt%.B_4_C	2.92	2.94	3.15
Sintering Temperature 475 °C and 15 wt%.B_4_C	3.05	3.15	3.27
Sintering Temperature 475 °C and 15 wt%.B_4_C	3.19	3.28	3.39
**9**	**Compaction Pressure** **300 MPa**	**Compaction Pressure** **400 MPa**	**Compaction Pressure** **500 MPa**
Sintering Temperature 575 °C and 15 wt%.B_4_C	2.97	2.96	3.20
Sintering Temperature 575 °C and 15 wt%.B_4_C	3.09	3.24	3.33
Sintering Temperature 575 °C and 15 wt%.B_4_C	3.23	3.31	3.45

**Table 2:** Effect of PM parameters on micro hardness at different compaction pressure (300, 400, and 500 MPa), different sintering time (1, 2, and 3 h), different sintering temperature (375, 475, and 575 °C), and different reinforcement weight percentages (5 wt%., 10 wt%., and 15 wt%.B_4_C).

**1**	**Compaction Pressure** **300 MPa**	**Compaction Pressure** **400 MPa**	**Compaction Pressure** **500 MPa**
	**Sintering Time 1 h**	**Sintering Time 2 h**	**Sintering Time 3 h**
Sintering Temperature 375 °C and 5 wt%.B_4_C	141.59	144.72	147.09
Sintering Temperature 375 °C and 5 wt%.B_4_C	145.70	147.92	149.88
Sintering Temperature 375 °C and 5 wt%.B_4_C	147.66	148.66	151.25
**2**	**Compaction Pressure** **300 MPa**	**Compaction Pressure** **400 MPa**	**Compaction Pressure** **500 MPa**
Sintering Temperature 375 °C and 5 wt%.B_4_C	137.9	145.27	147.82
Sintering Temperature 375 °C and 5 wt%.B_4_C	140.85	146.75	149.73
Sintering Temperature 375 °C and 5 wt%.B_4_C	147	154.35	159.18
**3**	**Compaction Pressure** **300 MPa**	**Compaction Pressure** **400 MPa**	**Compaction Pressure** **500 MPa**
Sintering Temperature 375 °C and 5 wt%.B_4_C	133.27	115.92	120.09
Sintering Temperature 375 °C and 5 wt%.B_4_C	167.30	135.24	145.75
Sintering Temperature 375 °C and 5 wt%.B_4_C	148.21	112.62	133.41
**4**	**Compaction Pressure** **300 MPa**	**Compaction Pressure** **400 MPa**	**Compaction Pressure** **500 MPa**
Sintering Temperature 375 °C and 10 wt%.B_4_C	138.97	122.75	116.32
Sintering Temperature 375 °C and 10 wt%.B_4_C	143.89	120.45	119.66
Sintering Temperature 375 °C and 10 wt%.B_4_C	146.71	118.85	122.02
**5**	**Compaction Pressure** **300 MPa**	**Compaction Pressure** **400 MPa**	**Compaction Pressure** **500 MPa**
Sintering Temperature 375 °C and 10 wt%.B_4_C	141.71	164.85	157
Sintering Temperature 375 °C and 10 wt%.B_4_C	148.32	165.11	162.53
Sintering Temperature 375 °C and 10 wt%.B_4_C	152.38	167	164
**6**	**Compaction Pressure** **300 MPa**	**Compaction Pressure** **400 MPa**	**Compaction Pressure** **500 MPa**
Sintering Temperature 375 °C and 10 wt%.B_4_C	148.52	129.25	136.06
Sintering Temperature 375 °C and 10 wt%.B_4_C	154.70	130.11	142.70
Sintering Temperature 375 °C and 10 wt%.B_4_C	158.71	132.41	149.82
**7**	**Compaction Pressure** **300 MPa**	**Compaction Pressure** **400 MPa**	**Compaction Pressure** **500 MPa**
Sintering Temperature 375 °C and 10 wt%.B_4_C	161.56	133.77	139.65
Sintering Temperature 375 °C and 10 wt%.B_4_C	163.87	145.69	150
Sintering Temperature 375 °C and 10 wt%.B_4_C	166.01	149.02	151.77
**8**	**Compaction Pressure** **300 MPa**	**Compaction Pressure** **400 MPa**	**Compaction Pressure** **500 MPa**
Sintering Temperature 375 °C and 10 wt%.B_4_C	154.84	161.32	167.02
Sintering Temperature 375 °C and 10 wt%.B_4_C	163.55	170.92	184.41
Sintering Temperature 375 °C and 10 wt%.B_4_C	174.20	178.61	182.66
**9**	**Compaction Pressure** **300 MPa**	**Compaction Pressure** **400 MPa**	**Compaction Pressure** **500 MPa**
Sintering Temperature 375 °C and 10 wt%.B_4_C	150.44	146	165.98
Sintering Temperature 375 °C and 10 wt%.B_4_C	159.14	153.76	169.08
Sintering Temperature 375 °C and 10 wt%.B_4_C	163	158.03	173

**Table 3:** Effect of PM parameters on compressive strength at different compaction pressure (300, 400, and 500 MPa), different sintering time (1, 2, and 3 h), different sintering temperature (375, 475, and 575 °C), and different reinforcement weight percentages (5 wt%., 10 wt%., and 15 wt%.B_4_C).

**1**	**Compaction Pressure** **300 MPa**	**Compaction Pressure** **400 MPa**	**Compaction Pressure** **500 MPa**
	**Sintering Time 1 h**	**Sintering Time 2 h**	**Sintering Time** **3 h**
Sintering Temperature 375 °C and 5 wt%.B_4_C	125.49	127.04	117.32
Sintering Temperature 375 °C and 5 wt%.B_4_C	132.53	131.48	109.77
Sintering Temperature 375 °C and 5 wt%.B_4_C	137.66	140	105.33
**2**	**Compaction Pressure** **300 MPa**	**Compaction Pressure** **400 MPa**	**Compaction Pressure** **500 MPa**
Sintering Temperature 475 °C and 5 wt%.B_4_C	130.66	137.74	142.55
Sintering Temperature 475 °C and 5 wt%.B_4_C	141	144.66	149
Sintering Temperature 475 °C and 5 wt%.B_4_C	156.03	160.93	165.53
**3**	**Compaction Pressure** **300 MPa**	**Compaction Pressure** **400 MPa**	**Compaction Pressure** **500 MPa**
Sintering Temperature 575 °C and 5 wt%.B_4_C	132.67	135.53	140.05
Sintering Temperature 575 °C and 5 wt%.B_4_C	142	147.75	152.90
Sintering Temperature 575 °C and 5 wt%.B_4_C	151.55	157.11	163
**4**	**Compaction Pressure** **300 MPa**	**Compaction Pressure** **400 MPa**	**Compaction Pressure** **500 MPa**
Sintering Temperature 375 °C and 10 wt%.B_4_C	108.09	107	106.03
Sintering Temperature 375 °C and 10 wt%.B_4_C	117.07	112.79	110.64
Sintering Temperature 375 °C and 10 wt%.B_4_C	121.88	120.41	116.22
**5**	**Compaction Pressure** **300 MPa**	**Compaction Pressure** **400 MPa**	**Compaction Pressure** **500 MPa**
Sintering Temperature 475 °C and 10 wt%.B_4_C	130.66	110.81	138.07
Sintering Temperature 475 °C and 10 wt%.B_4_C	135.04	122.66	145.98
Sintering Temperature 475 °C and 10 wt%.B_4_C	141.06	129.32	155.11
**6**	**Compaction Pressure** **300 MPa**	**Compaction Pressure** **400 MPa**	**Compaction Pressure** **500 MPa**
Sintering Temperature 575 °C and 10 wt%.B_4_C	144.23	139.44	136.06
Sintering Temperature 575 °C and 10 wt%.B_4_C	154.77	146.62	142.12
Sintering Temperature 575 °C and 10 wt%.B_4_C	159.03	148	145.88
**7**	**Compaction Pressure** **300 MPa**	**Compaction Pressure** **400 MPa**	**Compaction Pressure** **500 MPa**
Sintering Temperature 375 °C and 15 wt%.B_4_C	139.09	141	133.16
Sintering Temperature 375 °C and 15 wt%.B_4_C	143.22	147.19	136.42
Sintering Temperature 375 °C and 15 wt%.B_4_C	149.18	152.88	145.02
**8**	**Compaction Pressure** **300 MPa**	**Compaction Pressure** **400 MPa**	**Compaction Pressure** **500 MPa**
Sintering Temperature 475 °C and 15 wt%.B_4_C	151.12	126.42	147.33
Sintering Temperature 475 °C and 15 wt%.B_4_C	156.29	139.02	150.06
Sintering Temperature 475 °C and 15 wt%.B_4_C	164.33	148.04	157.83
**9**	**Compaction Pressure** **300 MPa**	**Compaction Pressure** **400 MPa**	**Compaction Pressure** **500 MPa**
Sintering Temperature 575 °C and 15 wt%.B_4_C	149.33	159.36	122.12
Sintering Temperature 575 °C and 15 wt%.B_4_C	156.88	167.41	135.81
Sintering Temperature 575 °C and 15 wt%.B_4_C	161.10	171	143.04

## Data Availability

Not applicable.
